# Supplementary material for: Directly imaging excited state-resolved transient structures of water induced by valence and inner-shell ionisation
Source: Nat Commun. 2023 Sep 5;14:5420. doi: 10.1038/s41467-023-41204-x (PMC10480213; doi:10.1038/s41467-023-41204-x)
Supplement: Supplementary file 1 — Supplementary information [file 41467_2023_41204_MOESM1_ESM.pdf]

Supplementary Information for "Directly imaging excited state-resolved transient structures of water induced by valence and inner-shell ionisation "

Zhenzhen Wang[1,+], Xiaoqing Hu[2,+], Xiaorui Xue[3,+], Shengpeng Zhou[1], Xiaokai Li[1], Yizhang Yang[1], Jiaqi Zhou[3], Zheng Shu[2], Banchi Zhao[1], Xitao Yu[1], Maomao Gong[4,5], Zhenpeng Wang[4,2], Pan Ma[1], Yong Wu[2,6*], Xiangjun Chen[4], Jianguo Wang[2], Xueguang Ren[3*], Chuncheng Wang[1*], and Dajun Ding[1*]

[1]Institute of Atomic and Molecular Physics and Jilin Provincial Key Laboratory of Applied Atomic and Molecular Spectroscopy, Jilin University, Changchun 130012, China
[2]Key Laboratory of Computational Physics, Institute of Applied Physics and Computational Mathematics, Beijing 100088, China
[3]School of Physics, Xi'an Jiaotong University, Xi'an 710049, China
[4]Hefei National Research Center for Physical Sciences at Microscale and Department of Modern Physic, University of Science and Technology of China, Hefei, Anhui, 230026, China.
[5]School of Physics and Information Technology, Shanxi Normal University, Xi' an 710119, China
[6]HEDPS, Center of Applied Physics and Technology, Peking University, 100871 Beijing, China.
[*]Correspondence and requests for materials should be addressed to Chuncheng Wang (email: ccwang@jlu.edu.cn) or to Yong Wu (email: wu yong@iapcm.ac.cn) or to (renxueguang@xjtu.edu.cn) or to Dajun Ding (email: dajund@jlu.edu.cn)
[+]these authors contributed equally to this work

Contents

I Supplementary Note 1 **2**
I.1 Energy correlation map and Dalitz diagram . . . 2
II Supplementary Note 2 **3**
II.1 Two-dimensional fitting procedure . . . 3
III Supplementary Note 3 **5**
III.1 Simulation details . . . 5
IV Supplementary Note 4 **6**
IV.1 All approximations used in present simulations . . . 6
V Supplementary Note 5 **8**
V.1 Stepwise summary of the theoretical results . . . 8
VI Supplementary Note 6 **19**
VI.1 Comparison between the measurements and simulations with different laser parameters . . . 19
VII Supplementary Note 7 **21**
VII.1 Experimental setup . . . 21
References **21**

Supplementary Notes

I Supplementary Note 1

I.1 Energy correlation map and Dalitz diagram

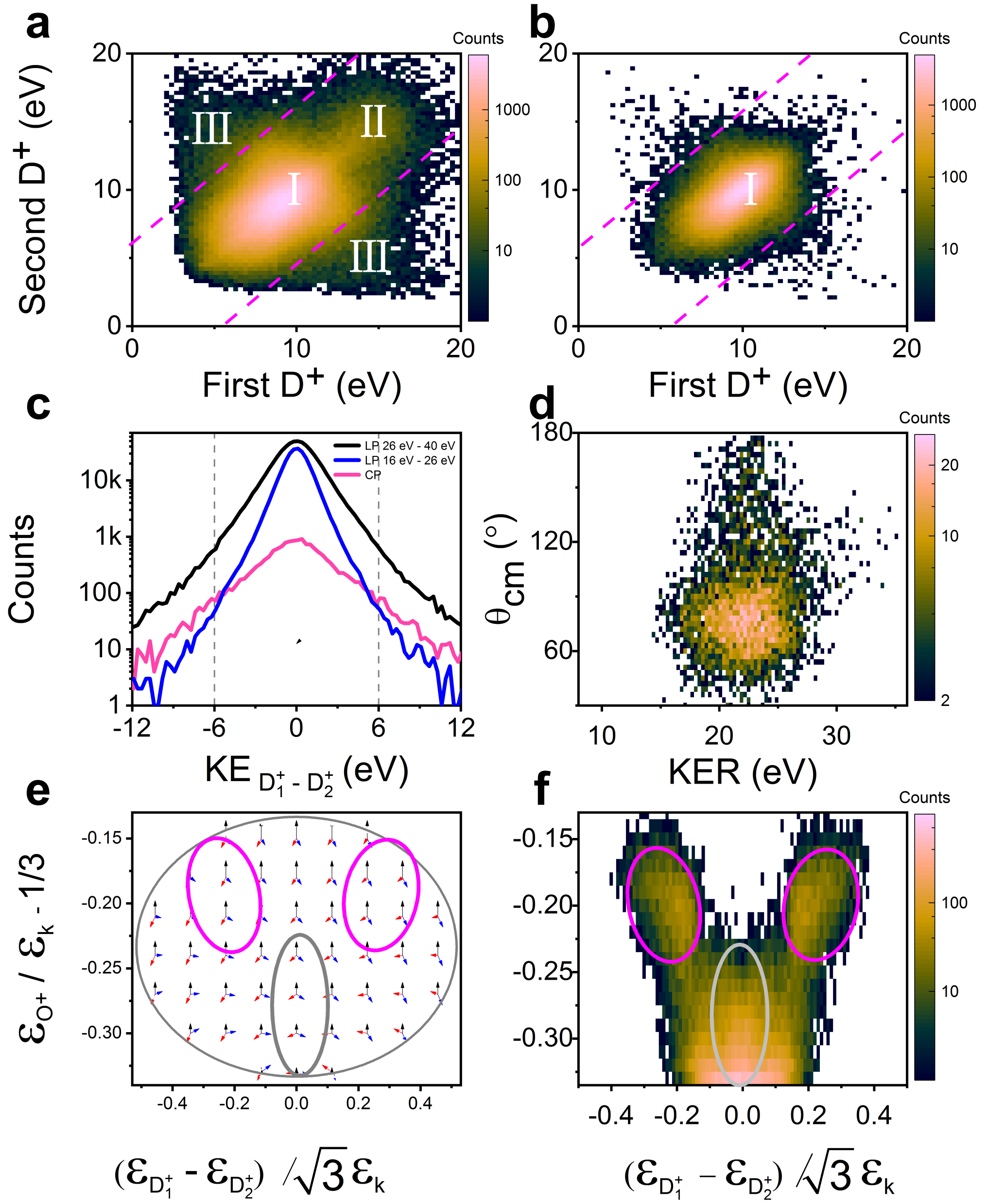

Supplementary Figure 1. Energy correlations of two D^+ and Dalitz diagrams. **a** and **b** present energy correlation maps of the two D^+ for linear polarisation (LP) and circular polarisation (CP), respectively, and the distributions with strong polarisation-dependence are labeled II and III, which originate from TERCE. The distributions beyond the dashed lines are named region III. **c** presented the one dimensional distributions of energy differences between two D^+ for linear and circular polarisation. The counts beyond $\pm$ 6 eV (indicated by the dashed lines) are further shown as the (θ_{cm}-KER) map for linear polarisation, which provides the distribution purely from the region III **d**. **e** simulated and **f** measured Dalitz diagrams for events from Fig. 1c in the main text, and the distribution from regions II and III are marked by the grey and pink solid circles, respectively.

The energy correlations between two D^+ are presented in Supplementary Figure 1a and b for linearly and circularly polarised light, respectively. The absence of regions II and III in the case of circularly polarised light suggests that the three-body electron recollision-assisted Coulomb explosion (TERCE) is the dominant mechanism. Region I and II are concentrated along the diagonal of the energy correlation map, which indicates that the two D^+ have a similar energy, and their geometries before CE are expected to be symmetrical. However, region III presents a distribution along the inverse diagonal, which suggests that the two D^+ are strongly correlated with larger energy differences. The energy differences of two D^+ in the case of the linearly and circularly polarised pulse are plotted in Supplementary Figure 1c, the majority of the counts in the case of circular polarisation locate at the relatively lower energy differences region (within ± 3 eV). In the case of linearly polarised pulse (kinetic energy range of 16 to 26 eV), there are more than 7000 events for larger energy differences (beyond ± 6 eV), which exceed the energy cut-off in the case of the circularly polarised pulse (see the pink dashed lines in Supplementary Figure 1b). We present those counts in Supplementary Figure 1d, which corresponds to the (θ_{cm}-KER) distributions of region III. Such a large energy difference of two D^+ in this region can only be formed by the CE of an asymmetrical geometry of D_2O. Furthermore, we present the Dalitz diagram of the events from Fig. 1c of the main text in Supplementary Figure 1f, where the distinct differences between regions II and III can be seen. The X- and Y-axies of the Dalitz diagram are defined as

$$X = (\varepsilon_{D_1^+} - \varepsilon_{D_2^+})/\sqrt{3}\varepsilon_K, \quad Y = \varepsilon_{O^+}/\varepsilon_K - 1/3 \tag{1}$$

where ε_K denotes the kinetic energy[1]. As indicated by the solid elliptical curves in the simulated Dalitz diagram (Supplementary Figure 1e), the central distribution (region II) centred at zero (X-axis) represents the symmetrical concerted CE, whereas the arm-like distributions located at ± 0.2 (region III) indicate that those events originate from concerted CE processes with asymmetrical geometry.

The counts in the region I are 1000 times higher than that in region II, thus it is not possible to directly extract the peak positions of different components in II through the fit. We need to pick up the events from TERCE in Fig. 1 a as described in the main text. As shown in Supplementary Figure 1, the events in region II have larger KER and kinetic energy of D^+, and the symmetric CE is dominant in this region, thus their kinetic energy difference between D^+ and O^+ should be also larger. In Supplementary Figure 2a and b, we present the correlation map of the kinetic energy difference of D^+ (D_1^+ or D_2^+) and O^+ for all the events in the case of circular and linear polarisation, respectively. Through the comparison of Supplementary Figure 2a and b and the one-dimensional distribution in Supplementary Figure 2c, the events in region II can be efficiently selected by setting the kinetic energy difference of D^+ and O^+ are larger than 12 eV (marked by the dashed line). The value of 12 eV is the crossing point of two curves in Fig. 2c, which is the starting point of the hump in the linearly polarised pulse and also close to the energy cut-off in circular polarisation. Using this condition, most of the events in the region I can be excluded. As shown in Supplementary Figure 2b, some counts in the region III are included by applying this condition, but those events can be excluded by setting the kinetic energy difference of two D^+ is smaller than 6 eV, as discussed in the first section. By combining these two conditions, we selected the majority of events in region II, and the (θ_{cm}-KER) distributions of those events are shown in Supplementary Figure 2d. Some events from the region I are still included, but that will not affect the determinations of the peak position of different components in region II with our two-dimensional fit procedure. We combined the events in Supplementary Figure 1d and Fig. 2d, and obtained the results shown in Fig. 1c in the main text.

II Supplementary Note 2

II.1 Two-dimensional fitting procedure

A two-dimensional (2D) multiple Gaussian peak fitting program was developed to fit the experimental data. The fitting function can be expressed as

$$\mathrm{f(x,y)} = \mathrm{a_0} + \sum_{i=1}^{n} a_i * exp(-(x-c_{x,i})^2/2*(\sigma_{x,i}^2) - (y-c_{y,i})^2/2*(\sigma_{y,i}^2) \tag{2}$$

Here, n is the assumed number of components, c and σ denote the peak position and full width at half maximum of the 2D Gaussian distributions, respectively. According to the principle of nonlinear least squares (NLS), several sets of parameters, which correspond to the local minima for the given initial values of the parameters, can satisfy the NLS. To determine the global minimum, we implemented the following improvements.

1. The parameters were assigned suitable initial values.
2. A set of parameters was obtained according to NLS. Its fitting error was recorded as the initial error of the global minimum, and these parameters were recorded as the optimized parameters.

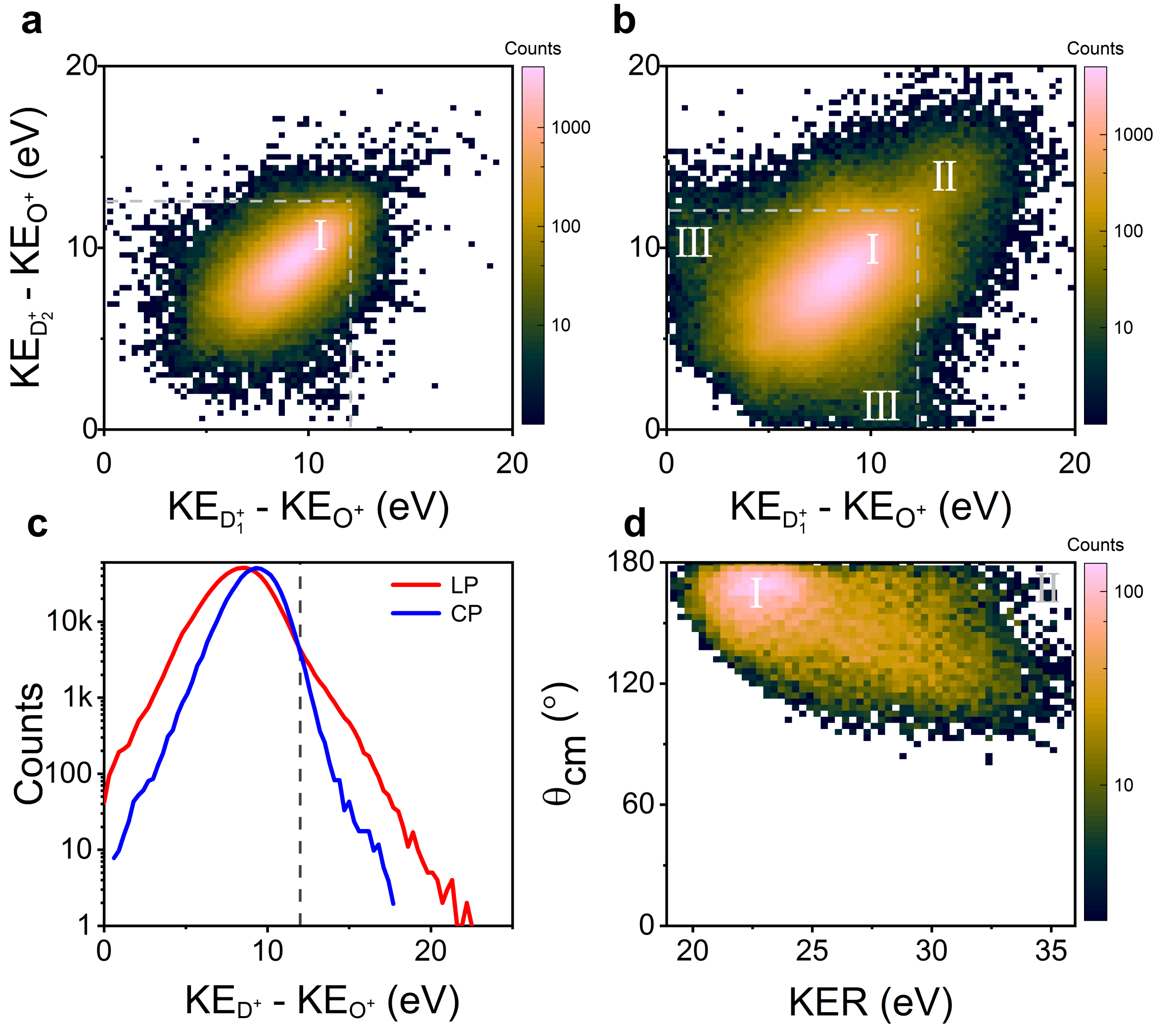

Supplementary Figure 2. Selection of the events from TERCE. **a** and **b** present the correlation map of the energy differences between the two D^+ and O^+ for the circular polarisation (CP) and linear polarisation (LP), respectively. The dashed lines stand for the value of 12 eV, which is used as a condition for selecting the events from region II. **c** presents their one-dimensional distributions of the energy differences between D^+ and O^+ for circular and linear polarisations. Two curves are normalized to the maximum and cross at 12 eV. **d** shows the (θ_{cm}-KER) distribution for the selected events after applying the conditions discussed in the text.

3. Some of the peak positions in the set of optimized parameters were reset to random values within the allowed range. These values were designated to be the initial values of the parameters for the next cycle of NLS. Then, the next NLS computation was performed.
4. A new set of parameters was obtained from the new NLS cycle, and a new fitting error was also obtained. If the new fitting error was smaller than the error in step 2, the present fitting error and the corresponding parameters were assumed to be the new value of the error and the new parameters for the global minimum. Then, the program proceeded to Step 3 for the next iterative cycle. The condition to stop the loop is that the assumed global minimum error is smaller than the value from the new loop or the number of the cycles is larger than the preset number.

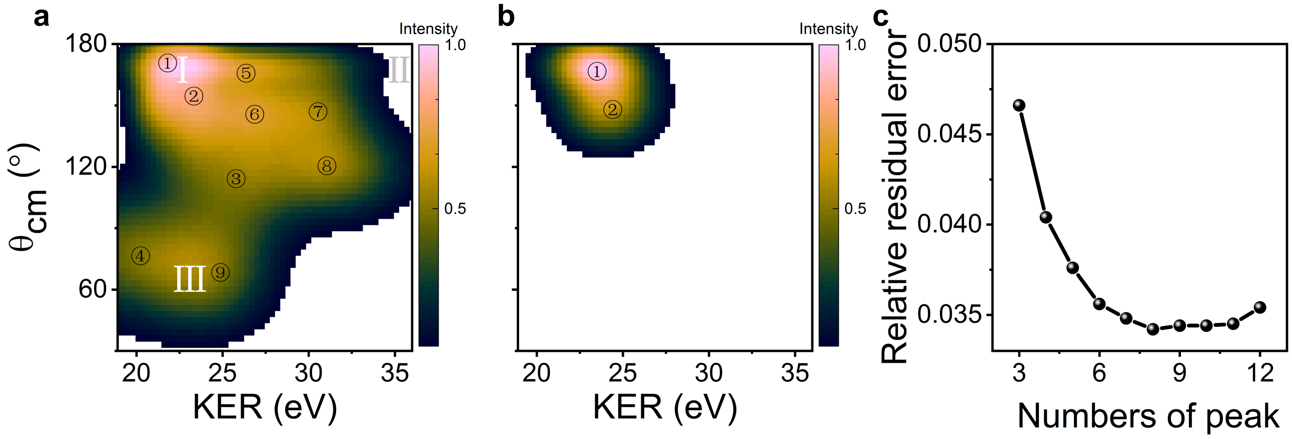

Supplementary Figure 3. The details of the 2D global fit. **a** 2D global fit of Fig. 1c in the main text with nine components, and the nine components in the region I marked with numbers. **b** 2D global fit of Fig. 1d in the main text with two components. **c** Summary of the residual errors of the 2D fit with an increasing number of components used in the fitting procedure.

Supplementary Figure 3a presents the outputs of our 2D fit for Fig. 1c in the main text. In the present case, the Fig. 1c in the main text can be fitted very well with nine components, and the Supplementary Figure 3b can be fitted with two components for circular polarisation. Two of the nine components are used to fit the distributions of region I, which are not relevant to the TERCE. The third and fourth components are very weak, and cannot be easily resolved in the raw data (Fig. 1c in the main text); thus, we refrained from discussing this component in the present work to avoid over-interpretation. The peak positions of the four components in region II are listed in Table 1 in the main text. We estimated the residual errors for assuming different numbers of components (from 3 to 12), as shown in Supplementary Figure 3c. The residual error $E_{res.}$ is defined as:

$$E_{res.} = \frac{1}{n}\sum_{i=1}^{n}(Y_i - \hat{Y}_i)^2 \quad (3)$$

where Y_i and $\hat{Y}_i$ represent the true and predicted values, respectively. We found that fitting with more than eight components can make residual errors small enough, and the errors reach a flat valley for 8 to 10 components.

III Supplementary Note 3

III.1 Simulation details

In this work, a custom-developed molecular dynamic method is used to simulate the three-body breakups of D_2O^{3+}. In contrast to the previous works, the present simulations can treat the whole nuclear dynamics processes of the sequential triple ionisation from D_2O to D_2O^{3+}. In this simulation, the molecular wave-packet is represented as $\Psi(r_1, r_2, \theta)\Phi(\alpha, \beta, \gamma)$, where r_1 and r_2 are the O-D bond lengths, θ is the bond angle, $\Psi(r_1, r_2, \theta)$ is the molecular vibrational function and $\Phi(\alpha, \beta, \gamma)$ is the orientational distribution. The initial D_2O is located at the X state of the neutral molecule with the ground vibrational state and isotropic orientational distribution, then evolves to the different charged states, D_2O^+, D_2O^{2+}, and D_2O^{3+} sequentially with the action of external laser and electron collision. Note that two widely used assumptions were applied in the present simulation:

i) The ionisations by intense laser pulse and electron impact satisfy the Franck-Condon principle, namely the nuclear vibrational wave-packets $\Psi(r_1, r_2, \theta)$ are kept frozen before and after the ionisations;
ii) The laser field can be considered as a perturbation interaction and only affects the orientational distribution $\Phi(\alpha, \beta, \gamma)$ without affecting the evolution of nuclear vibrational wave-packet $\Psi(r_1, r_2, \theta)$ along the potential energy surfaces. Its influences to the possible coupling of the vibrational states and electronic states are ignored (see details in the summary of approximations). Based on the two assumptions, the evolution of $\Psi(r_1, r_2, \theta)$ will only depend on the time intervals between two strong field ionisation, and the probabilities of strong field ionisation and electron collision ionisation determine the weight of the final $\Psi(r_1, r_2, \theta)$. Consequently, the present six-dimensional simulations are divided into two three-dimensional simulations. In

the first step, we simulated the evolution of $\Psi(r_1, r_2, \theta)$ from D_2O to D_2O^{3+} with different time intervals during ionisation values. In the second step, we calculated the weighting factors for different ionisation intervals by integrating the ionisation probability at different ionisation times with different orientations, where the change of molecular orientation distribution $\Phi(\alpha, \beta, \gamma)$ induced by laser are considered.

For the evolution of $\Psi(r_1, r_2, \theta)$ from D_2O to D_2O^{3+}, three different methods are used for treating the molecular ions D_2O^+, D_2O^{2+}, and D_2O^{3+}, respectively. In the case of D_2O^+, since the molecular wave-packet is mainly distributed on the bound vibrational state of molecular ions, $\Psi(t) = \sum_n C_n \psi(n) exp(-iE_n T)$ is applied, where $\psi(n)$ is the eigne wave function for D_2O^+, C_n is obtained by projecting the initial wave function to the $\psi(n)$ and T is the time interval between first and second ionisation. For the evolution along the potential energy surfaces of D_2O^{2+}, a full quantum time-dependent wave-packet method[2] is used to propagate the motion of the molecular wave-packet. For the third ionisation of electron re-collision, the maximal propagation time is fixed to 2.0 fs and the time step is set as 0.024 fs in this simulation. When the wave-packet is projected onto the D_2O^{3+}, the distributions of bond length and angle are recorded as $\Psi_T(r_1, r_2, \theta)$, where T is the time interval between single and double ionisation.

In order to obtain the weighting factors, the semi-classical MO-ADK[3] and Multicenter three-distorted-wave (MCTDW) methods[4] are used to calculate the ionisation probabilities for the first and second ionisation induced by intense laser pulses and the third ionisation induced by the electron re-collision, respectively. The final weighting factor W(T) can be written as

$$W(T) = \int dT_0 P_1(T, T_0) * P_2(T, T_0 + T) * P_3(T, T_0 + T + 2.0). \tag{4}$$

Where T is the time interval between single and double ionisations and $2.0\,fs$ is the time interval between double ionisation and triple ionisation induced by the electron re-collision ionisation. T_0 is the ionisation time of first single ionisation, T_0 + T and T_0 + T + 2.0 are the times of the double ionisation and electron re-collision induced triple ionisation, respectively. P_1, P_2 and P_3 are the total ionisation probabilities for three successive ionisations, in which we have considered the effect of molecular orientation on strong field ionisation as equation (5).

$$P = \int d\alpha d\beta d\gamma P(\alpha, \beta, \gamma) \Phi^2(\alpha, \beta, \gamma). \tag{5}$$

Where, $P(\alpha, \beta, \gamma)$ is the calculated ionisation probabilities for different molecular orientation. In present simulations, the initial distribution of molecular orientation $\Phi(\alpha, \beta, \gamma)$ is set as isotropic. Then, the full quantum time-dependent wave-packet method is applied to simulate the time-dependent $\Phi(\alpha, \beta, \gamma)$ under the influence of laser dipole polarisation interaction on molecular angular distribution. The polarisabilities used in the simulation can be obtained based on the *ab initio* calculations. We also consider the influence of ionisation on molecular orientation by

$$\Phi_a(\alpha, \beta, \gamma) = C * \sqrt{P(\alpha, \beta, \gamma)} \Phi_b(\alpha, \beta, \gamma). \tag{6}$$

Where $\Phi_b(\alpha, \beta, \gamma)$ and $\Phi_a(\alpha, \beta, \gamma)$ are the angular distribution before and after ionisations, respectively. C is the re-normalization coefficient.

After obtaining the distributions of bond length and angle $\Psi_T(r_1, r_2, \theta)$ of D_2O^{3+} and the corresponding weighting factor W(T), the initial distribution of Coulomb explosion simulation for D_2O^{3+} can be written as

$$D(r_1, r_2, \theta) = \int dT W(T) |\Psi_T(T, r_1, r_2, \theta)|^2. \tag{7}$$

A widely-used semi-classical trajectory method is adopted to simulate the three-body breakups of D_2O^{3+}. Totally 10^5 events are sampled based on the distribution D(r_1, r_2, θ). The maximal propagation time is $\sim$ 500 fs and the time step is $\sim$ 0.24 fs in the present simulations. Finally, we can obtain the momentum distribution of the three ions D^+, D^+, and O^+.

Besides, the average of the molecular wave-packets is treated as the molecular configurations at different ionisation times. The corresponding ionisation energies are set as the energy difference of the molecular wave-packet before and after ionisation. For the molecular orbital wave function required for MO-ADK and MCTDW calculations, high precision *ab initio* calculations are performed using the multi-configuration self-consistent field (MCSCF) method. In present simulations, the linearly polarised electric field E(t) is applied and $\mathbf{E(t)} = E_0 sin^2(\pi t/\tau) sin(\omega t + \phi)$, where E_0 is the peak field amplitude, ω is the carrier frequency, τ is the pulse duration, and ϕ is the carrier envelope phase. Note that the re-collision electron energy of 70.0 eV is adopted in the present calculations.

IV Supplementary Note 4

IV.1 All approximations used in present simulations

We simulated the TERCE process including the complex triple ionisation from D_2O to D_2O^{3+}, and the dynamics in three different directions need to be included, they are (1) the evolutions of nuclear wave-packets in different electronic states, (2) the

strong field tunnelling ionisation of molecule and (3) the impact ionisation from dication to trication by laser-induced returning electrons. The theoretical development in each direction is usually independent, and when we integrate those theoretical approaches together, all the approximations used in each direction need to be included in the current approach. Moreover, we also need to add new approximations to enable the combination of those approaches together. All approximations used in present simulations are listed in the following.

1): The ionisations by laser and electron collision both satisfy the Franck-Condon principle, i.e., the nuclear vibrational wave-packets $\Psi(r_1, r_2, \theta)$ are kept frozen before and after the ionisations. This approximation is widely used in the study of both strong field ionisation and the electron impact ionisation because the time scale of molecular vibration is much larger than that of ionisation.
2): The laser field can be considered as a perturbation interaction and only affects the orientational distribution $\Phi(\alpha, \beta, \gamma)$. The influence of the coupling of the vibrational states and electronic states via dipole transition has been ignored in TERCE, and thus the evolution of nuclear vibrational wave-packet $\Psi(r_1, r_2, \theta)$ along the potential energy surfaces is independent of the laser field. This approximation is justified as following: (i) The vibration time scale of molecules is usually significantly larger than the period of the laser optical, resulting in the cancellation of the work done by the oscillating fields to the molecular vibration. (ii) For the dipole transition from X to the A state via absorbing photon, we calculated the excited energy between X and A states. The energy difference between X and A states (D_2O^+) is 0.7 eV larger than the photon energy (1.55 eV for 800 nm pulse) (see Supplementary Figure 4), thus the resonance condition of dipole transition can not be met, and the probability of transition from X to A state is expected to be small. For the dipole transition from A state to X state via emitting photon, since the orientation-dependent ionisation rate shows that the O-D_2 axis of ionic molecule prefers to align to the direction of laser polarisation direction, which is perpendicular to the transient dipole moment direction. Hence, the probability of the transition from A to X state is also not significant. More importantly, it is found that the most probable interaction time between laser and X (or A) states is very short (within 10 fs) in the TERCE, and the short interaction time leads the contributions of coupling of X and A states to be less than 5% (estimated by simulations) in the present experimental condition (see Supplementary Figure 5). We simulated the (θ_{cm}-KER) distributions by including the coupling effects between X and A states in our model, where only the dipole transition occurs at the peak of the laser pulse is calculated. We found this coupling effect can play a minor role in TERCE by enhancing the distribution of larger θ_{cm} region (larger than 170°), however, the contributions from the direct tunnelling ionisation are still dominant. Thus we mainly focused on the nuclear dynamics triggered by the directly tunnelling ionisation in the present manuscript, and will explore the complex nuclear dynamics triggered by the photo-excitation in the future.

3): The MO-ADK method is used to calculate the ionisation yields of D_2O. This method is chosen for the following reasons: (i) high speed of calculation; (ii) accurate wave functions and ionisation energy of ionised orbital can be used. Here, the molecular orbital wave function and the corresponding ionisation energy are closely related to the evolution of molecular vibration wave-packet, which is a challenge to be considered by Time-dependent Hartree-Fock (TDHF) or Time-Dependent Schr*ö*dinger Equation(TDSE)[5]. To the best of our knowledge, this is one of the most recognized methods to calculate the strong field ionisation yield of simple molecules.

4): The electron-water collision ionisation cross sections are calculated by the MCTDW method. Here, the influence of direct distorted potential, polarisation potential and exchange potential in the distorted-wave calculation have been considered. Moreover, the effect of the multicenter nature of molecules on the continuous electron wave function is also considered. With these developments, the MCTDW method has been proven to be capable to simulate the low-energy electron impact ionisation probability of water ion[6].

5): The average of the molecular wave-packets is treated as the molecular configurations at different ionisation time. This approximation is widely used in the simulation of strong field ionisation and electron impact ionisation.

6): Only the ionisation at the peak of the electric field in each optical cycle is considered in the simulations. This approximation is used since the strong field ionisation yield exponentially depends on the amplitude of electric field in the laser pulse.

7): A semi-classical trajectory method is used to simulate the three-body breakups of D_2O^{3+}. This method is widely used and cheap in computation power for such ultrafast breakups dominated by coulomb potential. Its validity is also justified by well reproducing the measured (θ_{cm}-KER) distributions of the direct triple ionisation induced by the electron impact ionisation.

8): The influence of intensity volume effect of strong field ionisation on Coulomb explosion imaging is ignored because of

the limitation of computing resources. Moreover, the effects of varying ionisation energy with the evolution of vibration wave-packet is dominant to the TERCE relative to the influence of involving different laser intensities in the focus (see Supplementary Figure 14).

9): Considering the evolution of rotating wave-packet before and after ionisation through equation (5). So far, accurate theoretical method to calculate the effect of strong filed ionisation on the rotational wave-packet is still lacking, we can only use this rough approximation in our simulations. However, such approximation has little effect on the validity of the simulated fragmentation results because the rotation of D_2O molecules is not significant before the production of D_2O^{3+}, as can be seen in Supplementary Figure 11.

V Supplementary Note 5

V.1 Stepwise summary of the theoretical results

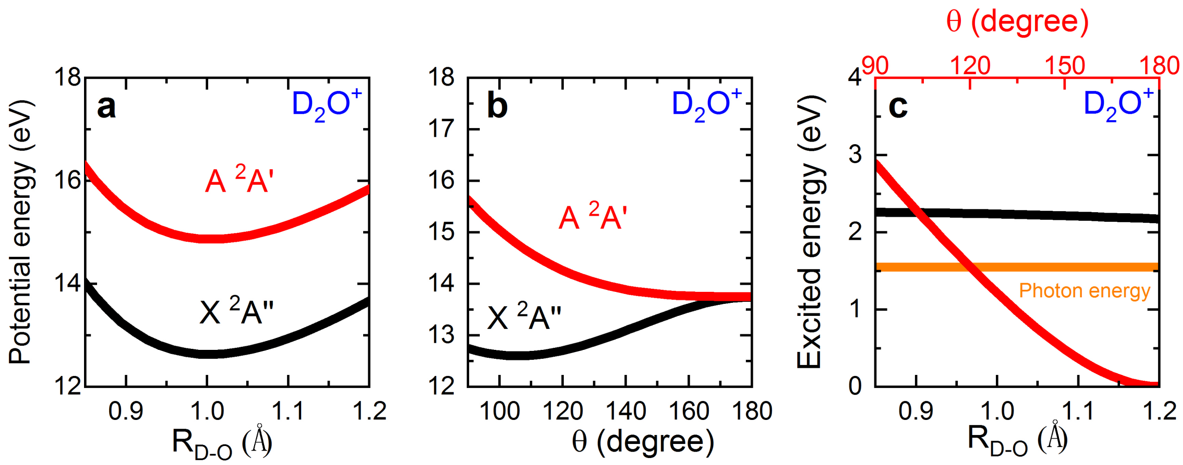

Supplementary Figure 4. Analysis of the photo-excitation between X and A states. The potential energy curves of ground (X) and excited (A) states of D_2O^+ are presented in **a** and **b**, and the comparison between their excited energies and photon energy are shown in **c**.

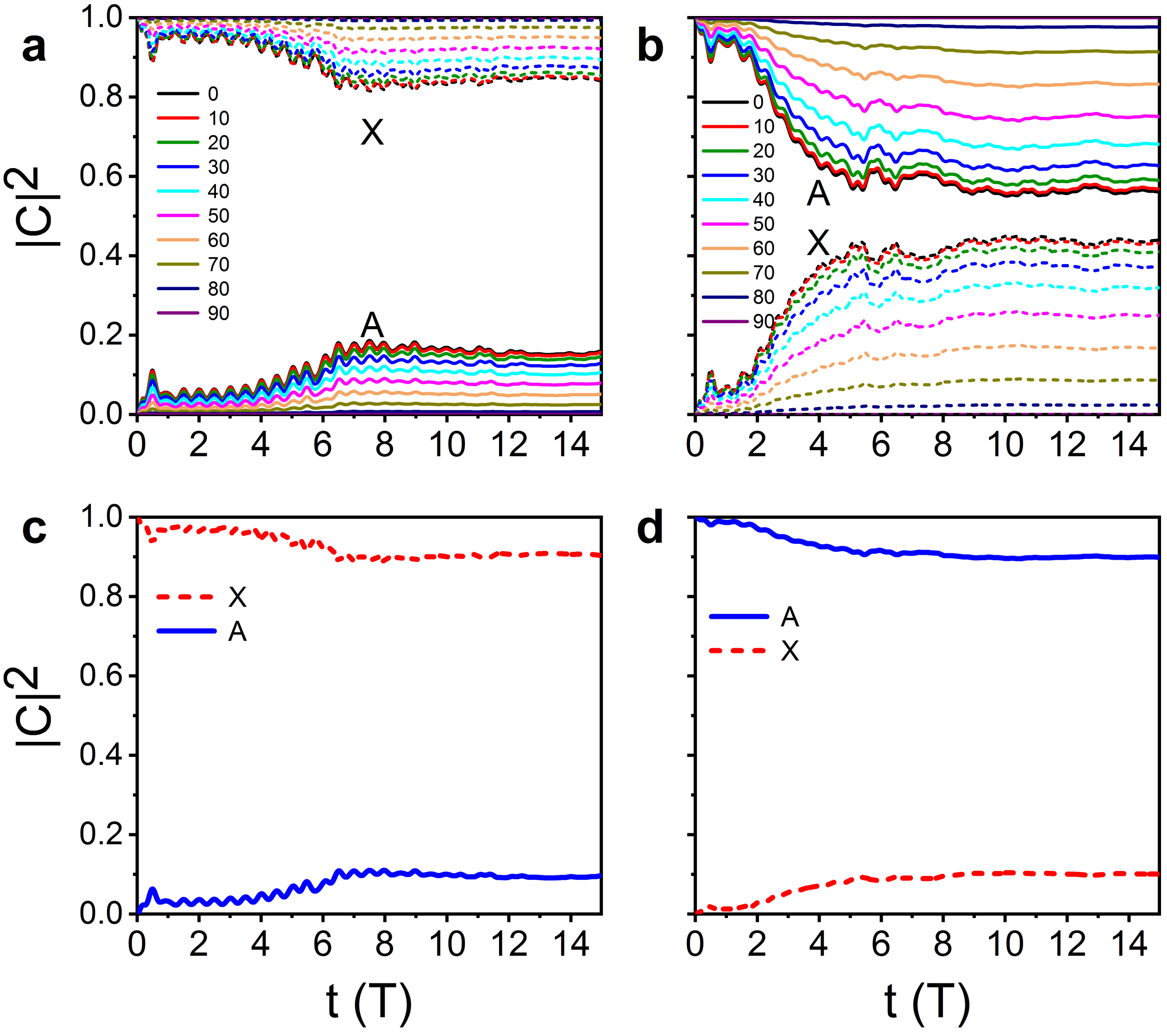

Supplementary Figure 5. Simulated time-resolved population transfers between X and A states through dipole transition process. **a** shows the results as the X state is initially populated. The time-resolved distributions of populations rely on the angle between the laser polarisation and the dipole moment (0° to 90°). **b** is the same as **a** but the A state is initially populated. **c** and **d** are the results of integrating all orientations. Here, the molecular orientation-dependent tunnelling ionisation yields are considered for both HOMO and HOMO-1 ionisation. The vertical axis represents the populations of A and X states, the time axis stands for the interaction time between molecule and laser pulse, T stands for the optical cycle (2.7 fs in our case).

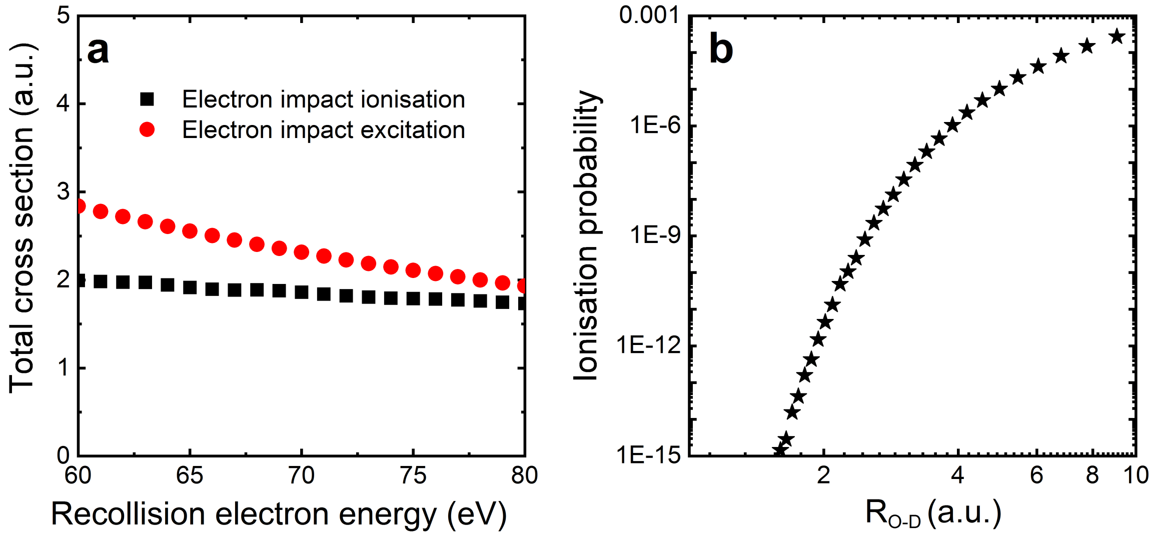

Supplementary Figure 6. Calculated cross sections and ionisation probability. **a** Calculated the total cross sections of electron impact excitation and electron impact ionisation. **b** presents the ionisation probability of dication after the electron impact excitation.

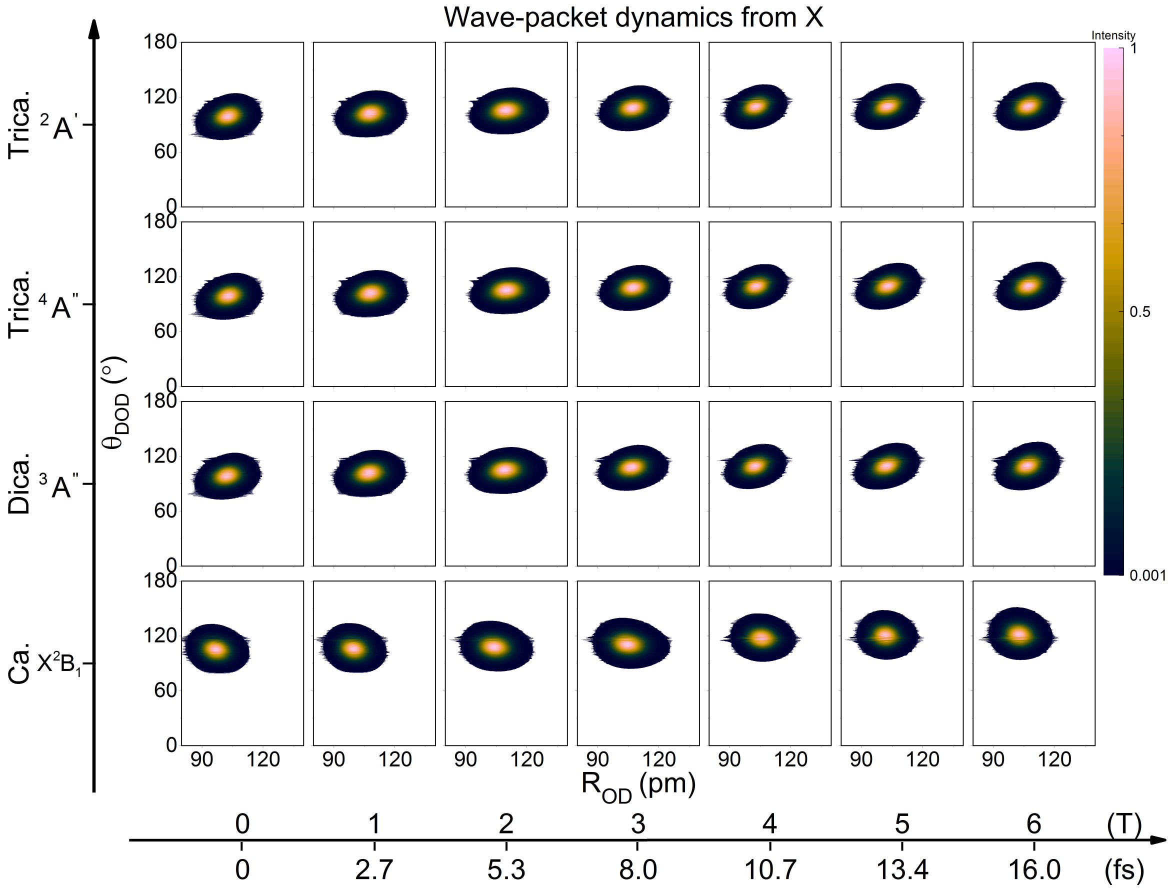

Supplementary Figure 7. Simulated time-dependent vibrational wave-packets dynamics for different ionisation stages. The lowest row from the bottom presents the vibrational wave-packet (WP) distributions along the X state at different time intervals, and are initiated by the tunnelling ionisation of HOMO. The second row from the bottom presents the WP distributions after the evolution of 2 fs in the dication. The third and fourth rows present the WP distribution of quartet and doublet states in the trication, respectively, The X axis stands for the time-delay between single ionisation and double ionisation. T stands for the optical cycle (2.7 fs in our case).

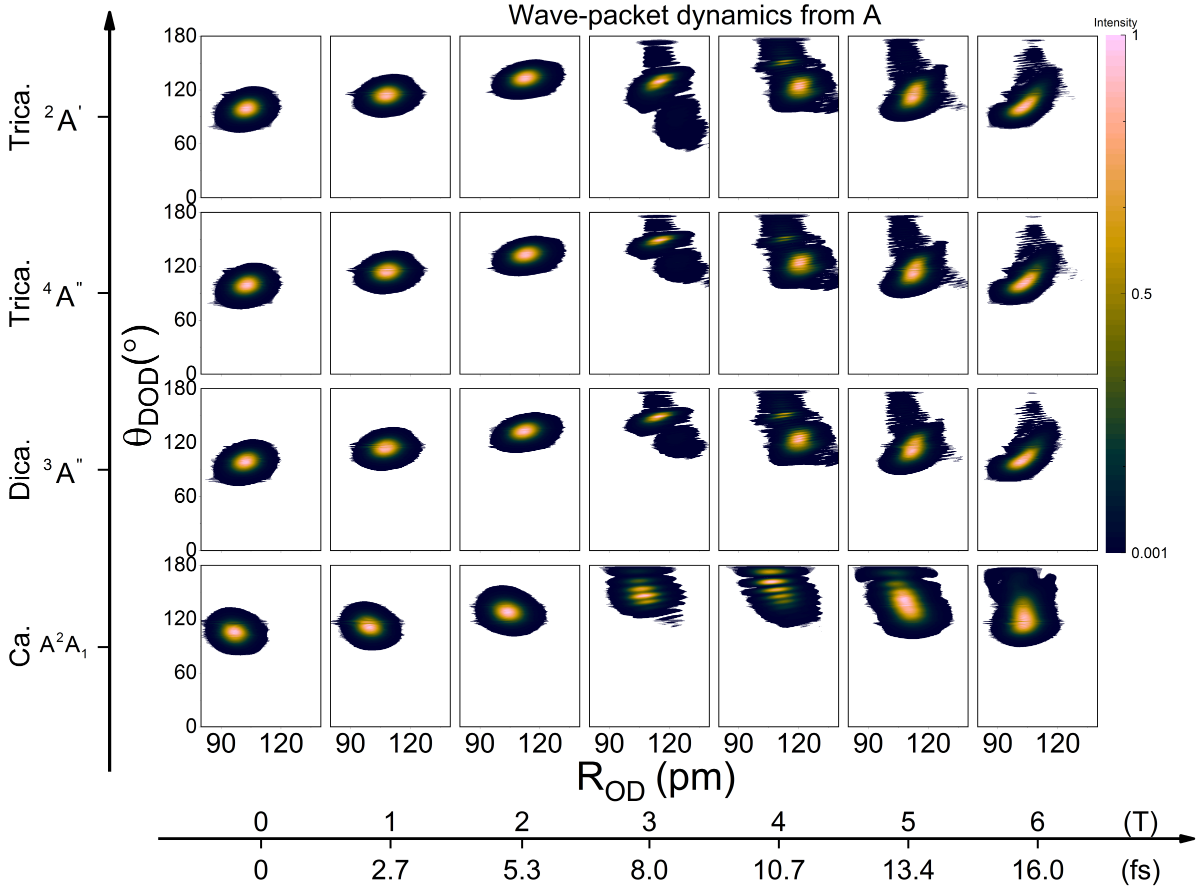

Supplementary Figure 8. Simulated time-dependent vibrational wave-packets dynamics for different ionisation stages. The lowest row from the bottom presents the vibrational WP distributions along the A state at different time intervals, and are initiated by the tunnelling ionisation of HOMO-1. The second row from the bottom present the WP distributions after the evolution of 2 fs in the dication. The third and fourth rows presents the WP distribution of quartet and doublet states in the trication, respectively, The X axis stands for the time-delay between single ionisation and double ionisation. T stands for the optical cycle (2.7 fs in our case).

1): The evolutions of $\Psi(r_1, r_2, \theta)$ from D_2O to D_2O^{3+} have been simulated with different time intervals between single and double ionisation. Here, the ionisation at the peak of each optical cycle is considered, and the calculated results are presented in Supplementary Figures 7. and 8, which provide the wave-packets evolution starting from the cationic X and A states, respectively. The x axis stands for the ionisation time interval. For the X state, the distributions of wave-packets are well localised in the direction of θ_{DOD}, and the wave-packets expand and shrink periodically along R_{OD}. For the A state, the wave-packets first expand both along θ_{DOD} and R_{OD}, and then start to shrink for larger time intervals, which are significantly different from the X state. Furthermore, at each time interval, the wave-packets are projected to the ground state of dication, and the electron-recollision induced triple ionisation occurs after 2 fs (electron recollision time). The evolutions of wave-packets along the potential energy surface of the ground state for dication during 2 fs are calculated and the results are presented in the second row of Supplementary Figures 7 and 8. For both case of ionisation from X and A states, the wave-packets extend to a larger value of R_{OD} for all time-intervals, while the distributions along θ_{DOD} oscillate as increasing the time intervals. Finally, the wave-packets after the evolution of 2 fs are projected to the trication and are saved as $\Psi_T(r_1, r_2, \theta)$. The third and fourth rows present the wave-packets distributions for the quartet and doublet states before the Coulomb explosion. They are almost the same as the second row for both X and A states. Thus the reconstructed transient structure from the TERCE can well reflect the geometry of dication. By considering the evolutions of 2 fs along the ground state of dication, the transient structure of X and A states can be revealed.

2): Calculate the average geometry of the time-dependent molecular wave-packets and the corresponding dipole moments and polarisabilities. In supplementary Figure 9, the calculated average values of R_{OD} and θ_{DOD} for different time intervals are

presented.

3): To calculate the final weight factor $W(T)$ according to equation (4), we first calculate the ionisation probabilities for different ionisation stages, including the single and double ionisation induced by intense laser pulses and the triple ionisation induced by the electron re-collision. All the possible combinations of ionisation times for triple ionisation during the laser pulse are considered. In supplementary Figure 10a, the calculated ionisation probabilities of HOMO-1 from neutral to cation at instant of 46.66 fs are presented as an example. From cation to dication, the ionisation probabilities for two time intervals (0 fs and 8.0 fs) are presented in supplementary Figure 10b and c, respectively. After the evolution of 8 fs along the A state, the ionisation probabilities increase by 12 times than that of 0 fs.

4): Simulate the evolution of rotational wave-packet $\Phi(\alpha,\beta,\gamma)$ using the time-dependent wave-packet method, where the required parameters have been obtained in step 2. α, β, and γ represent the angles between the laser field direction and the molecular plane, H-H and O-H2 directions, respectively. For neutral D_2O, since the polarisabilities in three directions are similar (9.35 a.u. for H-H direction, 9.98 a.u. for the axis perpendicular to the molecular plane and 9.61 a.u. for O-H_2 direction[7]), the current laser pulse will not significantly change the orientation of molecules. Supplementary Figure 11 presents the time-dependent angular distribution for orientation angle α (corresponding the direction with the largest polarisability). It can be seen that the molecular orientation will not change significantly before the molecule is ionised in our case.

5): Calculate the final weighting factor $W(T)$ for different time intervals according to equation (4) and equation (5). In Supplementary Figure 12a and c, the weighting factor $W(T)$ from X state of cation to the doublet and quartet states of trication are presented for different time intervals between single and double ionisation, respectively. The $W(T)$ reaches maxima as the time interval is two optical cycles for both cases. Similarly, the W(T) from A state of cation to the doublet and quartet states of trication are presented in Supplementary Figure 12b and d, and the maxima appear at three optical cycles of time intervals for both pathways. Here, both the influence of molecular orientation and ionisation at different instants are included in the calculations. As shown in Supplementary Figure 9a, for the cationic X state, the change of R_{OD} after evolutions of two optical cycles is indicated by the vertical black dashed arrows, and the change of R_{OD} during the electron recollision is indicated by the vertical red solid arrow. For the cationic A state, the change of R_{OD} after evolutions of three optical cycles is indicated by the vertical blue dashed arrows, and the change of R_{OD} during the electron recollision is indicated by the vertical pink solid arrow (see (c)); the change of θ_{DOD} after evolutions of three optical cycles is indicated by the dashed vertical line (see (d)).

6): Calculate the statistical bond length and bond angle distribution of D_2O^{3+} after the electron recollision ionisation by equation (7). The results for the channels from X and A state have been shown in Supplementary Figs. 13a and b, respectively.

7): Simulate the three-body breakups of D_2O^{3+} based on the distribution in the Supplementary Figure 13. Here, the semi-classical trajectory method is used to simulate the three-body breakups of D_2O^{3+}. Totally 10^5 events are sampled based on the distribution D(r_1, r_2,θ). The maximal propagation time is $\sim$ 500 fs and time step is $\sim$ 0.24 fs in the present simulations. Finally, we can obtain the momentum distributions of the three ions D^+, D^+, and O^+ for the different ionisation time intervals. The simulated results are shown as region II in Fig. 1e in the main text.

Besides, for the three-body Coulomb explosion induced by 200 eV electron impact, the interaction time of electron collisions is in attosecond regime, and the neutral molecules are directly populated into highly charged states, thus its simulations are different from that used for TERCE. However, for the breakup of D_2O^{3+}, the same semi-classical trajectory method is used to simulate the momentums of D^+ and O^+ for both electron impact ionisation and TERCE. Considering that the energy of the incident electron reaches 200 eV, the ground state and several excited states of trication are included in the simulation of electron impact ionisation. For the breakups of autoionisation state, the calculated orbital ionisation energy shows that only the D_2O^{2+} ($2a_1^{-2}$) state can contribute the three-body breakups with KER of approximately 30 eV. Since the potential energy surface of $2a_1^{-2}$ is also strongly repulsive, the semi-classical trajectory method is also suitable for simulating its breakup. We simulated the (θ_{cm}-KER) distribution of fragments after autoionisation to trication at different times. Finally, by comparing the simulated KER with the experimentally measured value, we can obtain the time of autoionisation and the values of θ_{cm}.

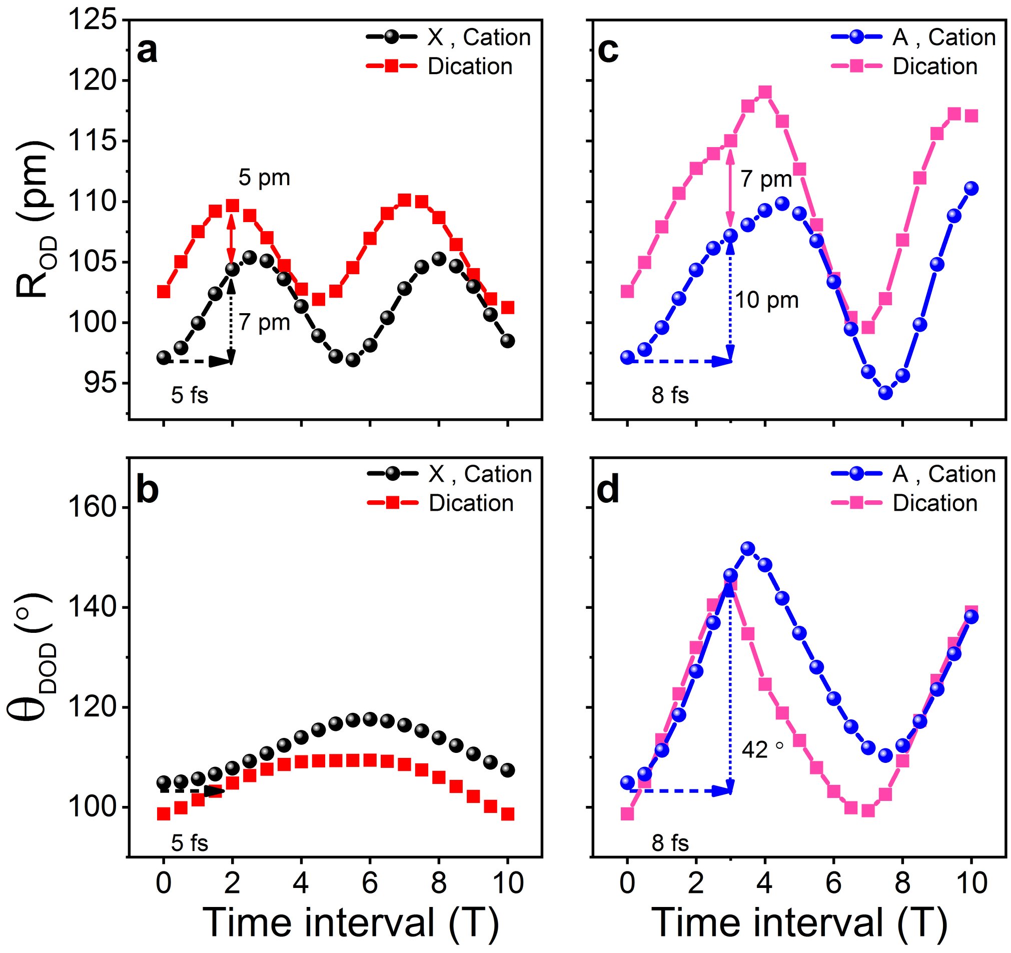

Supplementary Figure 9. Calculated average R_{OD} and θ_{DOD} of the time-dependent vibrational WP for D_2O^+ and D_2O^{2+} at different time intervals. **a** and **b** show the results starting from the X state, **c** and **d** show the results starting from the A state. The inserted numbers stand for the changes of R_{OD} and θ_{DOD} at the time intervals of two and three optical cycles for X and A states, respectively, T stands for the optical cycle (2.7 fs in our case).

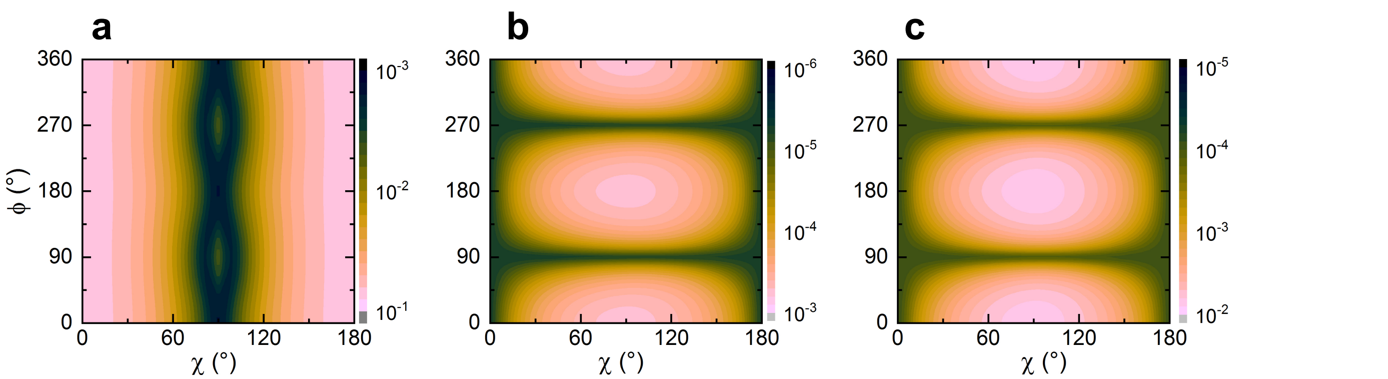

Supplementary Figure 10. Ionisation probabilities for the single and double ionisation induced by intense laser pulses. **a** presents the ionisation probabilities of HOMO-1 orbital for neutral D_2O with t = 46.66 fs; **b** presents the ionisation probabilities of HOMO orbital for D_2O^+ (A state) with t = 46.66 fs (time interval is 0 fs), where the bond length is 96.5 pm and bond angle is 104.5° and the ionisation energy is 24.75 eV; **c** presents the ionisation probabilities of HOMO orbital for D_2O^+ (A state) with t = 54.66 fs (time interval is 8.0 fs), where the bond length is 114.2 pm and bond angle is 106.5° and the ionisation energy is 22.75 eV. The ionisation probability increases by 12 times when the vibration WP evolves 8 fs in the A state.

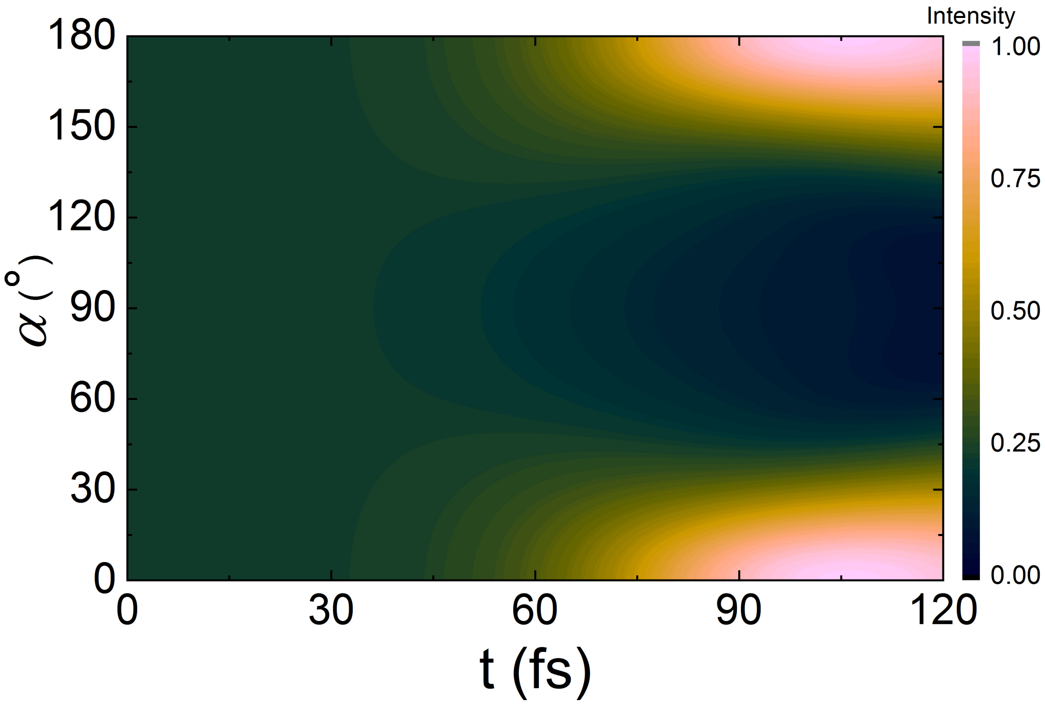

Supplementary Figure 11. Time-dependent angular distribution for different orientation angles (α) of neutral D_2O under the influence of the laser field, where α is the angle between the laser field and the axis in perpendicular to the molecular plane.

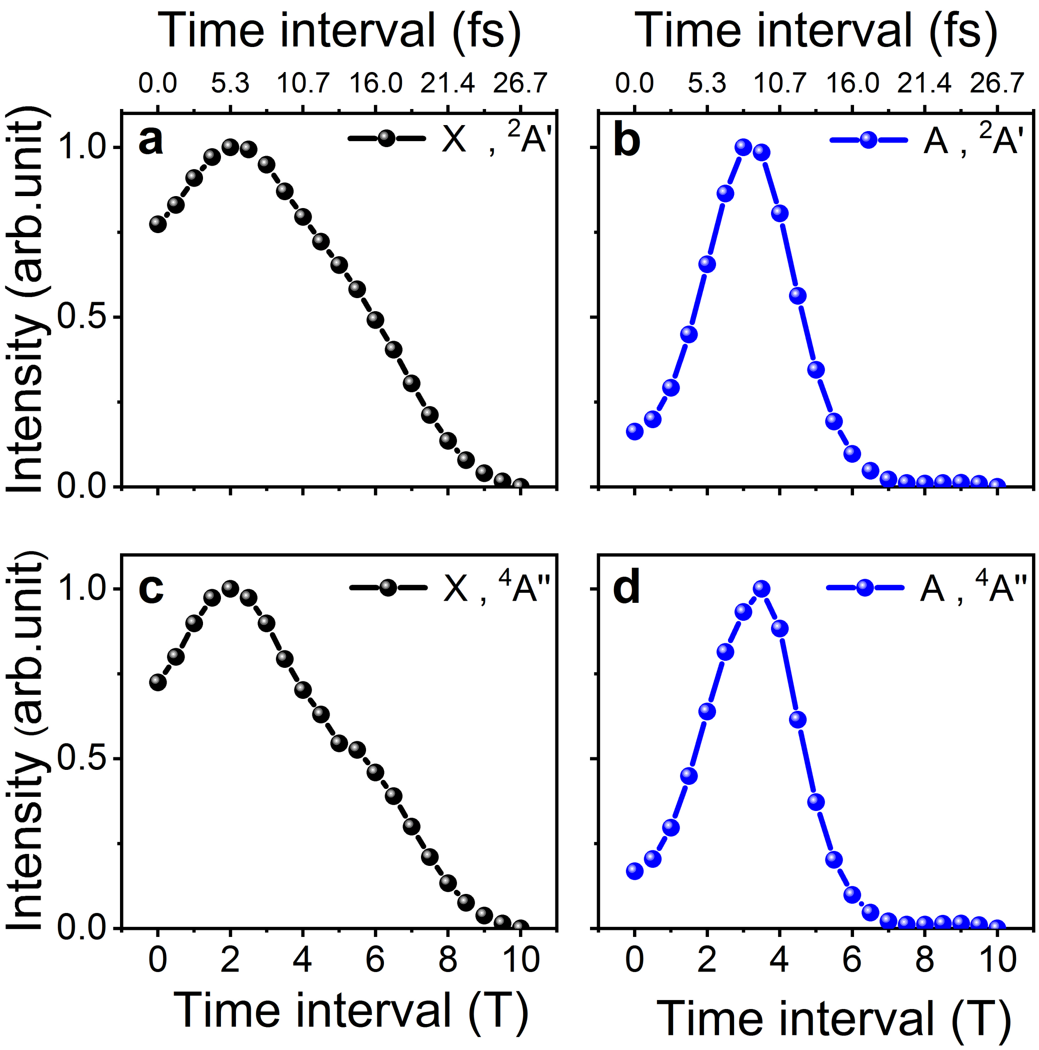

Supplementary Figure 12. The weighting factors ($W(T)$) for different ionisation intervals. **a** and **c** stand for the distributions from cationic X state to the doublet and quartet states, **b** and **d** are the same but from the cationic A state, T stands for the optical cycle (2.7 fs in our case).

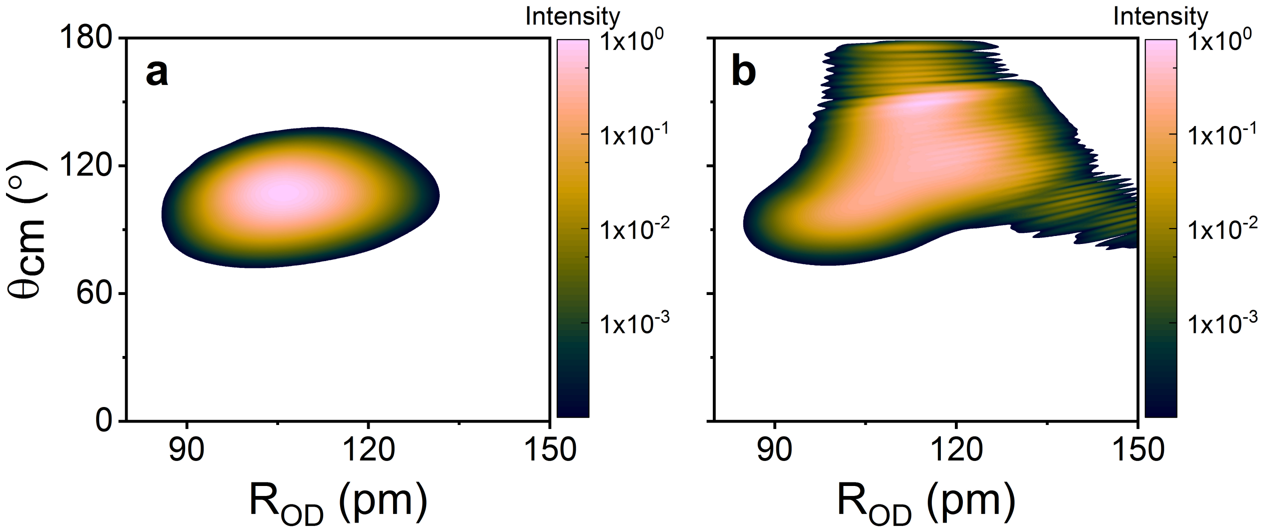

Supplementary Figure 13. The calculated distribution of (R_{OD}, θ_{DOD}) of D_2O^{3+} after considering the weighting factors before three-body Coulomb explosion. **a** and **b** stand for the WP distributions starting from cationic X and A states, respectively.

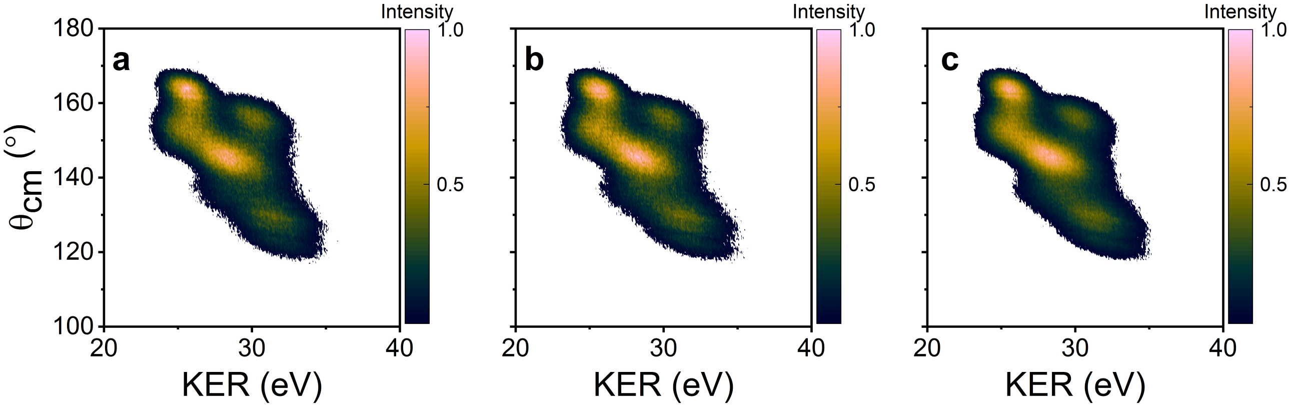

Supplementary Figure 14. Simulated (θ_{cm}-KER) distributions of the three-body CE channel induced by laser with different intensities. **a**, 100 TW/cm^2, **b**, 250 TW/cm^2 and **c**, 300 TW/cm^2. The distributions are normalized to the maximum value of the distribution.

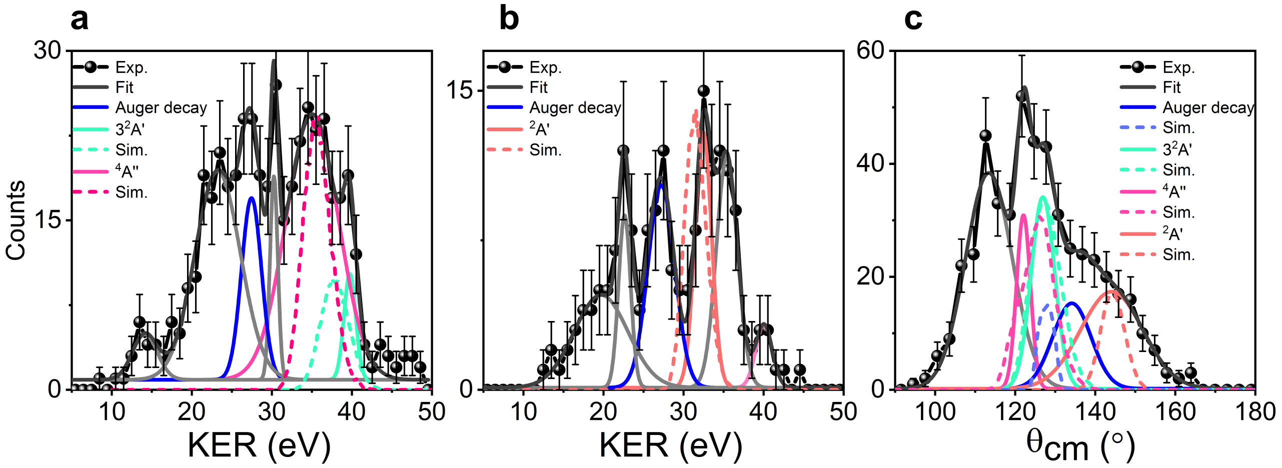

Supplementary Figure 15. One-dimensional distribution of KER and θ_{cm} of Coulomb explosion induced by the electron impact ionisation. **a** and **b** present the KER distribution as the θ_{cm} are in the range of (0°-132°) and (132°-150°) for the Fig. 4a in the main text, respectively. **c** show the distribution of θ_{cm} in the KER range of (25-50 eV). The statistical errors are presented in **a** to **c**. The black solid curve is the total fit of the experimental results (Exp.). The three components from the vertical triple ionisation are shown as the red, green and orange solid curves. One component from the Auger decay is shown as a blue solid curve. The distributions of KER and θ_{cm} from the simulation (Sim.) are inserted as the dashed curves for comparison.

VI Supplementary Note 6

VI.1 Comparison between the measurements and simulations with different laser parameters

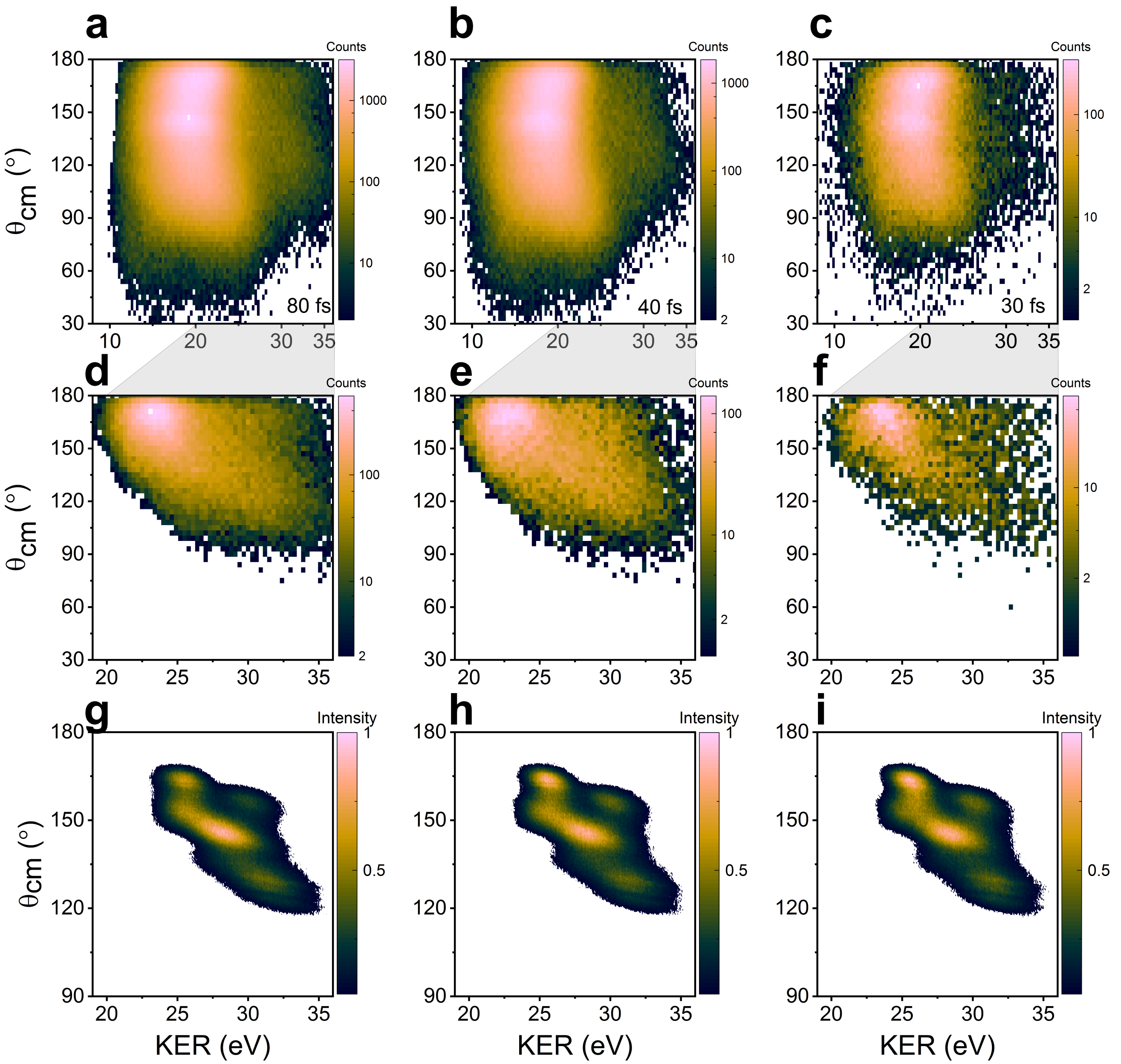

Supplementary Figure 16. (θ_{cm}-KER) distributions of the measurements with different pulse durations. **a** to **c** present the measured (θ_{cm}-KER) distributions and the selected events mainly from TERCE are shown in **d** to **f**. The total counts in **c** are less than other conditions. **g** to **i** are the simulated (θ_{cm}-KER) distributions with the same pulse durations as the measurement.

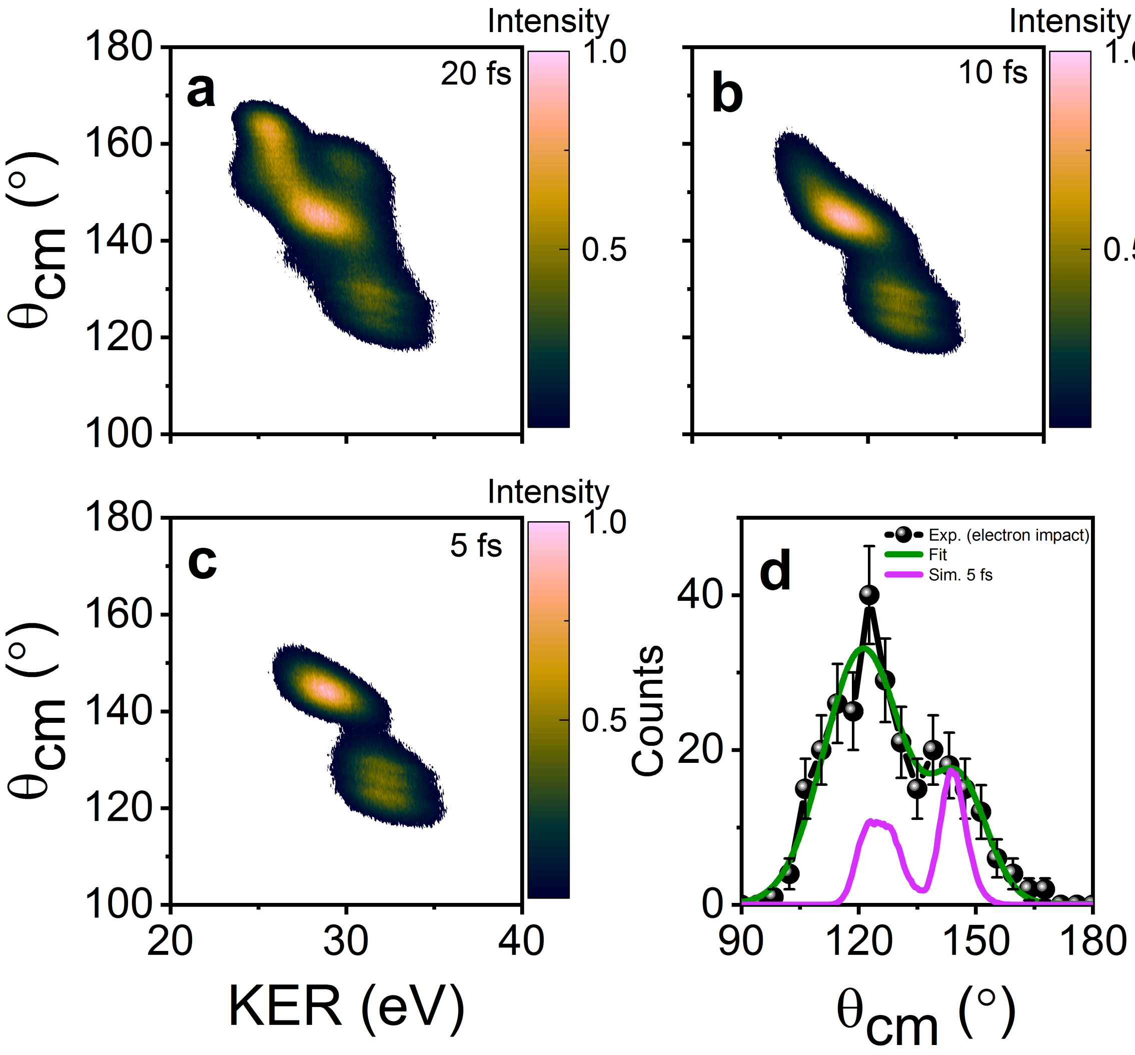

Supplementary Figure 17. (θ_{cm}-KER) distributions of TERCE ionised by the laser pulses with pulse durations shorter than 20 fs. **a** to **c** simulated (θ_{cm}-KER) distribution of TERCE for the pulse duration of 20 fs, 10 fs and 5 fs. **d** presents one-dimensional distributions of θ_{cm} for the two dominant components (KER: 30 eV - 39 eV) in the electron impact measurement and the simulated result of TERCE with pulse duration of 5 fs. The statistical errors are given.

We measured (θ_{cm}-KER) distributions from TERCE with different pulse durations including 30 fs, 40 fs and 80 fs, and we simulated the (θ_{cm}-KER) distributions from TERCE in a broad range of pulse durations, covering from 5 fs to 80 fs. The direct comparisons between the measurements and simulations for pulse durations of 30 fs, 40 fs and 80 fs are presented in Supplementary Figure 16, a good agreement can be reached and the distributions are overall similar for the pulse durations in this range. These results confirm the validity of our simulations. The simulated (θ_{cm}-KER) distributions with pulse durations of 5 fs, 10 fs and 20 fs are presented in Supplementary Figure 17, the dramatic shrinking to the smaller angle in the distributions of θ_{cm} can be seen as pulse duration decreases. According to our assignments in the main text, the observed changes in θ_{cm} originate from that the bending motion in the A state of cation is frozen by the ultrashort laser pulse, especially for the pulse duration of 5 fs. Interestingly, the direct vertical triple ionisation induced by the electron impact can also fully freeze the motion along the cations, and the distribution of θ_{cm} for this channel is presented and compared with the simulation, as shown in Supplementary Figure 17d. The peak positions of the two main components are quantitatively the same between the measurement and simulation, which are (122°, 144°) and (125°, 144°), respectively. For the distributions of the KER, the peak value of TERCE driven by a 5 fs laser pulse is smaller than that induced by the electron impact ionisation, which originates from the bond stretching motion along dication state during the electron re-collision process (2 fs). Those results suggest the temporal evolution of the wave-packet along the cation can be effected by the pulse durations. The comparison between the simulations of TERCE and the measurement with electron impact ionisation can also verify the validity of our

simulation approach.

VII Supplementary Note 7

VII.1 Experimental setup

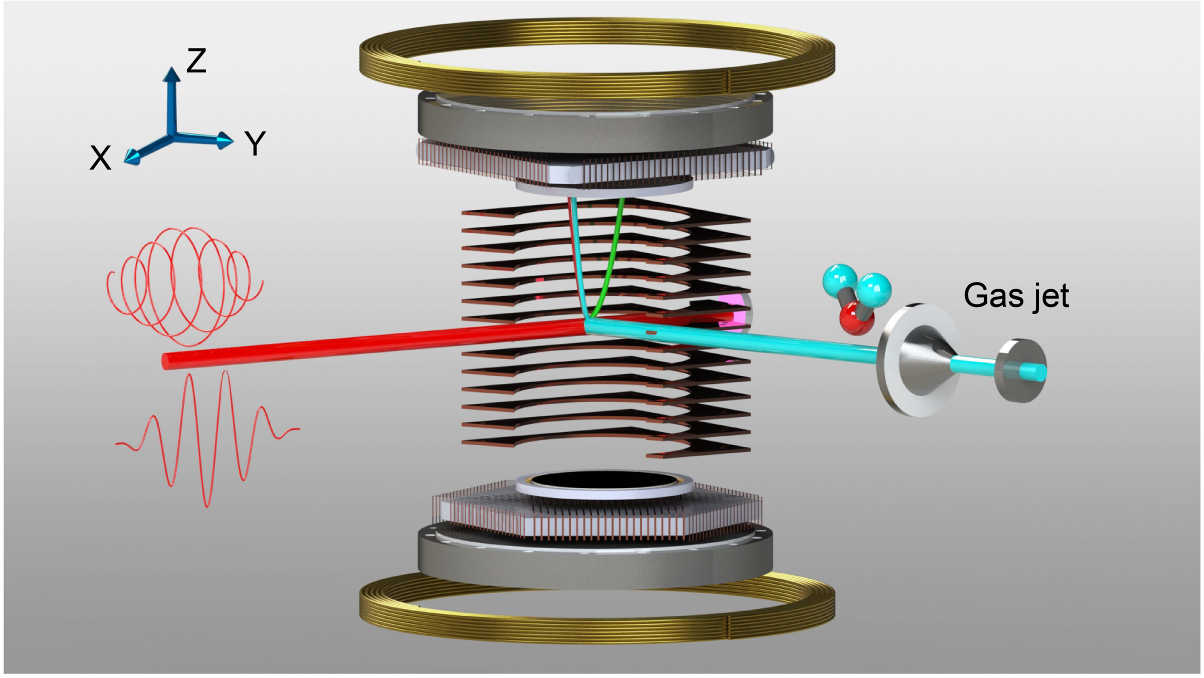

Supplementary Figure 18. Schematic diagram of the experimental setup that was used for TERCE. The D_2O molecules are introduced into the chamber via supersonic expansion and irradiated by the linearly and circularly polarised laser pulses. The three ions produced via the Coulomb explosion are detected in coincidence by the microchannel plate and delay-line detector and their three dimensional momentums can be obtained.

References

1. Neumann, N. *et al.* Fragmentation Dynamics of CO_2^{3+} Investigated by Multiple Electron Capture in Collisions with Slow Highly Charged Ions. *Phys. Rev. Lett.* **104**, 103201 (2010).
2. Hu, X., Jia, C., Xu, T., Wu, Y. & Wang, J. Full quantum time-dependent simulations for the two-body breakups of H_2Ar^{2+} and N_2Ar^{2+}. *Phys. Rev. A* **106**, 012814 (2022).
3. Gong, M. *et al.* Multicenter three-distorted-wave approach to three-dimensional images for electron-impact-ionization dynamics of molecules: Overall agreement with experiment. *Phys. Rev. A* **98**, 042710 (2018).
4. Knowles, P. J. & Werner, H.-J. An efficient second-order MC SCF method for long configuration expansions. *Chem. Phys. Lett.* **115**, 259–267 (1985).
5. Zhang, B., Yuan, J. & Zhao, Z. Dynamic core polarization in strong-field ionization of co molecules. *Phys. Rev. Lett.* **111** (2013).
6. Gong, M. *et al.* Multicenter three-distorted-wave approach to three-dimensional images for electron-impact-ionization dynamics of molecules: Overall agreement with experiment. *Phys. Rev. A* **98**, 042710 (2018).
7. Christiansen, O., Gauss, J. & Stanton, J. F. Frequency-dependent polarizabilities and first hyperpolarizabilities of CO and H_2O from coupled cluster calculations. *Chem. physics letters* **305** (1999).
